# Bioaccumulation of rare earth elements and trace elements in different tissues of the golden grey mullet (*Chelon auratus*) in the southern Caspian Sea

**DOI:** 10.1007/s10653-023-01593-w

**Published:** 2023-06-19

**Authors:** Shima Bakhshalizadeh, Adeleh Rostamzadeh Liyafoyi, Rafael Mora-Medina, Nahúm Ayala-Soldado

**Affiliations:** 1 Department of Marine Science, Caspian Sea Basin Research Center, University of Guilan, Rasht, Iran; 2 Department of Fisheries, Faculty of Natural Resources, University of Tehran, Karaj, Iran; 3 Department of Anatomy and Comparative Pathology and Toxicology, Faculty of Veterinary Medicine, University of Córdoba, Córdoba, Spain

**Keywords:** Bioaccumulation, Emerging pollutants, Fish, Trace elements, Rare earth elements

## Abstract

Rare earth elements are essential for modern life, although they are also classified as emerging pollutants. Currently, fish studies on these elements are very limited in general, but, with regard to the Caspian Sea, there is no reference to them at all. For this reason, our objective was to determine the concentrations of these elements in the golden grey mullet (*Chelon auratus*) and to contrast its bioaccumulation patterns with those of arsenic, cadmium, mercury and lead. For that purpose, 20 fish were caught in the southern part of the Caspian Sea. Heavy rare earth element concentrations were higher than light ones and the terbium levels were very high, probably due to anthropogenic contamination. The intestine tissue gave the highest concentrations, which could be indicative of a very low gastrointestinal absorption. For both rare earth and trace elements, muscle was the tissue that accumulated the least, despite which, cadmium and lead levels in muscle were of concern.

## Introduction

Rare earth elements (REEs) are a group of seventeen elements, fifteen of which correspond to lathanides, from lanthanum (La) (*Z* = 57) to lutetium (Lu) (*Z* = 71), in addition to scandium (Sc) (*Z* = 21) and yttrium (Y) (*Z* = 39) (Wu et al., 2022). All of these elements share similar physicochemical properties and are found naturally in the earth’s crust (Lachaux et al., 2022; Zhuang et al., 2017), except for promethium (Pm), which does not have stable isotopes (Elkina and Kurushkin 2020). The REEs are never encountered in a pure form since they are majority or minority components of a wide variety of minerals, which are found in ashalides, carbonates, oxides, phosphates and silicates (Dostal, 2017). These elements are generally split into two groups: light REEs (LREEs),which include La, cerium (Ce), praseodymium (Pr), neodymium (Nd), Pm, samarium (Sm), europium (Eu) and Sc; heavy REEs (HREEs), which comprise gadolinium (Gd), terbium (Tb), dysprosium (Dy), holmium (Ho), erbium (Er), thulium (Tm), ytterbium (Yb), Lu and Y. LREEs are generally considered to be more soluble than HREEs (Gonzalez et al., 2014; Hidaya and Abidin 2018).

Currently, REEs play an essential role in modern life due to their generalized technological implications, especially in the areas of energy, digital technologies, the nuclear industry and medical applications (Kegl et al., 2020; Pagano et al., 2019). It is probably because of their limited industrial use in the past that REEs have not been paid the necessary scientific attention by environmental and public health researchers. Despite this, these elements are classified as emerging pollutants since they are not legislated in humans or in the environment they are not monitored regularly, and their toxicity mechanisms are poorly understood (Gwenzi et al., 2018). This contrasts with other elements, like arsenic (As), cadmium (Cd), mercury (Hg) and lead (Pb), which are regularly monitored, are subject to legislation, and whose principal toxicological properties are well defined (IARC, 2012; Järup, 2003; European Commission, 2006).

The Caspian Sea is the largest enclosed body of water in the world, with a surface area of about 370,000 km^2^. It is located between Asia and Europe and borders five countries; Russia, Kazakhstan, Turkmenistan, Iran and Azerbaijan (Lattuada et al., 2019). Based on its geophysical characteristics, the Caspian Sea can be divided into three areas; the north, with a maximum depth of 30 m and a salinity under 10 g/L, and the middle and southern areas with a salinity close to 13 g/L, and depths of 800 m and of 1000 m, respectively (Kosarev, 2005; Lattuada et al., 2019). The Caspian Sea has abundant fishing, mineral, gas and oil resources. In recent decades, this area has been heavily affected by anthropogenic activity due to the large volume of debris and sewage poured daily into its coastal zones, the pollution in the rivers that flow into it and the waste from oil refineries (Bakhshalizadeh et al., 2022; Lattuada et al., 2019; Madani et al., 2022).

Fish are often employed as trace metal pollution indicators in the marine ecosystem, since they occupy high trophic levels and are an important food source for humans (Agah et al., 2008). The feeding habits of the golden grey mullet, *Chelon auratus*, belonging to the Mugilidae family, are characterized by a regular contact with the sediment, often extended to the whole water column. Therefore, it is a valuable bioindicator species for monitoring water contaminants (Cappello et al., 2016). Successfully introduced into the Caspian Sea from the Black Sea in the 1930’s (Naderi et al., 2017; Whitfield et al., 2012), it is one of the most valuable species in the Caspian sea, and its demand has increased in the past few years. The total catch golden grey mullet along its southern coast has been established at being approximately 4300 tonnes per year (Bahmani et al., 2011).

Due to the shortage of information on the presence of REEs in the Caspian Sea, one of the aims of our study was to determine their concentrations in the golden grey mullet and how they were ordered, i.e. from the most to the least abundant, by, comparing them with the normal values of these compounds in the earth’s crust. Also, another of the objectives was focussed on evaluating the REE bioaccumulation pattern in the fish’s different tissues to assess the differences from the bioaccumulation patterns of trace elements (As, Cd, Hg and Pb), that are frequently monitored.

## Material and methods

### Study area and sample collection

**Fig. 1:**
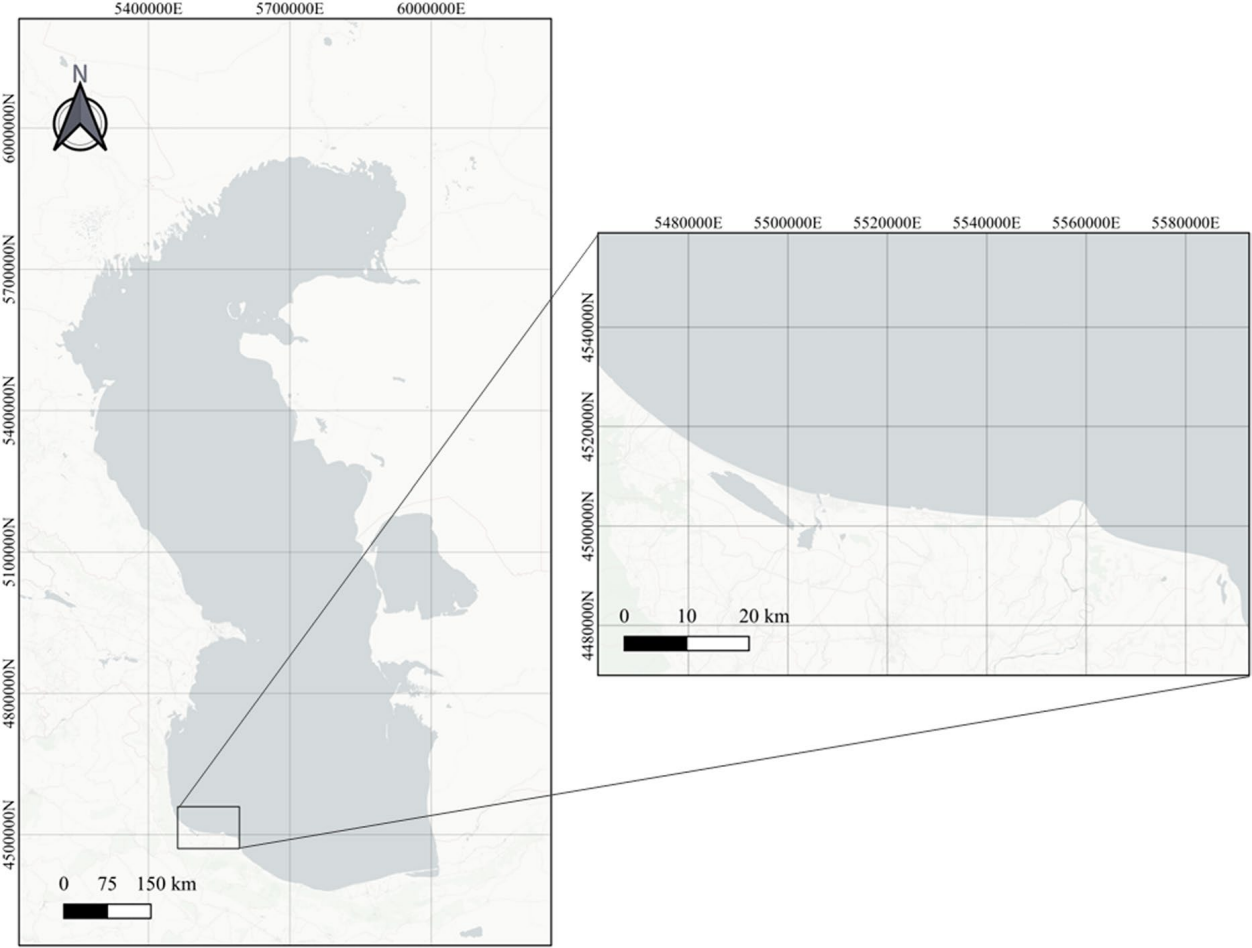
Localization of the sampling area in the southern Caspian Sea

A total of 20 golden grey mullets with an average weight of 385 ± 156 g, a length of 33.8 ± 7.24 cm and aged between 3 and 5 years, were caught randomly in the south of the Caspian Sea, on the Bandar Anzali (Iran) coast, as shown in Fig. 1. The fish were caught in nets, euthanized by concussion and kept on ice until sampled.

Before collecting samples from the fish, the animals were cleaned externally with distilled water to remove any dirt or substance that could pollute them. Subsequently, they were laid on their right side and both opercula were removed with some scissors. Next, three incisions were made; the first one from the anal fin up to the bottom end of the gill, the second one through the mean longitudinal line to the edge of the gills and the third one between the final points of the previous incisions. After these cuts had been made, intestine, liver, kidney and gill tissue were sampled. In addition, the fish were skinned in order to obtain samples of muscle tissue. A portion of appproximately 5 g of each tissue of interest was collected and weighed on an analysis scale. The instruments used (scissors, tweezers and scalpels) were washed in 1% nitric acid before taking each sample. The samples were frozen at − 80 °C until analysis. The whole process was performed in accordance with the European regulations for the protection of animals used for scientific purposes (European Parliament, 2010).

### REEs and trace elements analysis

After defrosting them, the tissue samples were homogenized and digested. Specifically, 1 g of sample from each tissue was digested in an acid solution composed of 10 mL of nitric acid (65% HNO_3_, Merck, Suprapur) and 5 mL of hydrogen peroxide (30% H_2_O_2_, Merck, Suprapur) heated on a plate at 200 °C. After complete digestion, the contents were decanted into a Falcon tube and diluted to 30 mL with deionized water (Milli-Q Millipore; resistivity 18.2 MΩ/cm). The analysis blanks were processed in the same way, and the concentrations were determined by using standard solutions prepared in the same acid matrix.

The following REEs: La, Ce, Pr, Nd, Sm, Eu, Gd, Tb, Dy, Ho, Er, Tm, Yb, Lu, Sc and Y, and the trace elements: As, Cd, Hg and Pb were quantified by an inductively coupled plasma mass spectrometer (ICP-MS) (Agilent 8900—Agilent Technologies, Palo Alto, CA, EE. UU.). The recovery of the analytical methods was verified by employing the certified reference materials DORM-4 (fish protein certified reference material for trace metals from National Research Council Canada) for trace elements and certified reference materials REE-1 (rare earth elements, zirconium and niobium from Natural Resources Canada). In accordance with Yang et al. (2016), the limits of detection (LOD) and quantification (LOQ) of each element were calculated through the analyte concentration that corresponded to three and ten times the standard deviation of ten independent blank measurements, respectively. The recovery values for the analytes examined ranged between 97 and 104%. The linearity test showed r^2^ values of above 0.997 for the elements analysed. In the case of REEs, extremely low concentrations were found.

### Data analysis

The statistical analysis of the data was made with version 25 SPSS software. To determine the concentration of REEs in the Caspian Sea, we added together the concentrations of these elements obtained in the different tissues of each fish. Based on these data, we also calculated the percentage of each REE over the total. Furthermore, ∑REEs, ∑LREEs and ∑HREEs were calculated in agreement with MacMillan et al. (2017), i.e. the LREEs included La, Ce, Pr, Nd, Sm, Eu and Sc, and the HREEs Gd, Tb, Dy, Ho, Er, Tm, Yb, Lu and Y. The ∑LREEs/∑HREEs were reckoned in order to estimate the ratio between LREEs and HREEs, in general, and for each tissue. We used the median for all the calculations since the data showed a wide variability.

In addition, Levene and Kolmogorov–Smirnov tests were applied to check homoscedasticity variances and data normality, respectively. However, both assumptions were violated, so it was decided to use a nonparametric method, i.e. the Kruskal–Wallis nonparametric H test, to determine statistically significant differences between tissues for each element and for ∑LREEs and ∑HREEs. The Kruskal–Wallis H test assesses the differences between three or more independently sampled groups on a single, non-normally distributed continuous variable (McKight & Najab, 2010). We also applied the Bonferroni correction as it is a multiple comparison method in pairwise analysis, but it is important to point out that the latter is a much more conservative method than other statistical analyses (Chen et al., 2017), which ensures that the differences found are representative. In all the cases, a value of *p* < 0.05 was taken as being significant.

## Results and discussion

### Concentration of REEs in the Caspian Sea

Table 1 shows the descriptive statistics of the REEs found in golden grey mullet in the Caspian Sea, together with the abundance of REEs in the earth’s crust, following Dushyantha et al. (2020). The order of the concentrations in the fish, based on the percentage of each element over the total of REEs, was as follows: Y (47.3%) > Nd (22.6%) > Tb (6.64%) > La (5.95%) > Ce (4.13%) > Gd (3.26%) > Sc (2.29%) > Pr (2.27%) > Dy (2.26%) > Sm (1.92%) > Er (0.64%) > Eu (0.32%) > Ho (0.18%) > Lu (0.10%) > Tm (0.10%) > Yb (0%). It should be noted that Y was the most abundant REE by far in this species, but we could not identify the source of this element. Future studies will probably be able to explain this. The order of accumulation obtained in the fish differs from that of their abundance in the earth's crust, which is as follow: Ce (35.8%) > La (16.5%) > Nd (14.4%) > Y (11.2%) > Sc (7.47%) > Pr (3.78%) > Sm (2.50%) > Gd (2.13%) > Dy (2.08%) > Er (1.22%) > Yb (1.17%) > Eu (0.53%) > Ho (0.44%) > Tb (0.37%) > Lu (0.16%) > Tm (0.16%).

**Table 1:** Descriptive statistics of the REEs determined in golden grey mullet (Chelon auratus) in the southern area of the Caspian Sea compared to REEs present in the earth's crust

REEs	Element	Z^a^	Mean (pg g^−1^)	DS (pg g^−1^)	Median (pg g^−1^)	Fish^b^ (%)	W REEs^c^ (ng g^−1^)	World^d^ (%)
LREEs	La	57	833	1715	184	5.95	31,000	16.5
	Ce	58	843	2142	128	4.13	67,000	35.8
	Pr	59	274	538	70.1	2.27	7100	3.78
	Nd	60	2875	5716	697	22.6	27,000	14.4
	Sm	62	239	476	59.3	1.92	4700	2.50
	Eu	63	41.4	84.7	9.72	0.31	1000	0.53
	Sc	21	332	762	70.7	2.29	14,000	7.47
HREEs	Gd	64	453	915	101	3.26	4000	2.13
	Tb	65	920	1890	204	6.64	700	0.37
	Dy	66	319	658	70.0	2.26	3900	2.08
	Ho	67	27.0	55.7	5.83	0.18	830	0.44
	Er	68	87.6	179	20.0	0.64	2300	1.22
	Tm	69	11.4	22.5	3.21	0.10	300	0.16
	Yb	70	44.5	99	0	0	2200	1.17
	Lu	71	10.6	21	3.38	0.10	310	0.16
	Y	39	7239	12,251	1460	47.3	21,000	11.2
∑LREEs			5438		1217	39.5	151,800	81
∑HREEs			9112		1868	60.5	35,540	19
∑REEs			14,550		3086	100	187,340	100

^a^Atomic number

^b^Percentage of each element over REE total in golden grey mullet

^c^REEs abundance in the earth’s upper crust (Dushyantha et al., 2020)

^c^Percentage of each element over total REE abundance in the earth’s upper crust

Data shown are the sum of the different tissues

As can be observed in Table 1, all the samples collected from the fish logically contained much lower REE concentrations than those found on the earth’s surface. However, it should be noted that Tb, which is one of the scarcest elements in the world (Gao et al., 2022), was the third most abundant REE, which suggests that its strong presence is a product of human pollution. In this regard, the Caspian Sea is under a constant threat from contamination, and oil extraction is one of its main sources (Ghasemi et al., 2019). Alshahri and El-Taher (2018) ascertained that people living near an oil refinery could be at a high risk of exposure to Tb, among other elements. Therefore, it has been suggested that the high Tb concentrations in golden grey mullet could originate from this contamination.

It is important to point out the lack of any studies on REE concentrations in fish in the Caspian Sea. In modern sediments, Maslov et al. (2016) reported concentrations of some REEs all over this sea. In the samples obtained in the southern area, they established a different order of concentrations to ours (Ce > La > Nd > Y > Sc), and it can be seen that the first places are occupied by LREEs. LREEs are also much more abundant in the earth’s crust (Dushyantha et al., 2020), specifically, ∑LREEs represents 81% of ∑REEs, as shown in Table 1. In our study, the ∑HREEs/∑LREEs were fixed at 1.53 in the golden grey mullet, i.e. more HREEs are accumulated than LREEs, which is also influenced by the large amount of Y detected. Squadrone et al. (2022) also found a greater presence of HREEs. In their study, the ∑HREEs/∑LREEs were of 7.20 and 36.3 in spotted dogfish blood and liver samples, respectively. However, Wang et al. (2022) and Yang et al. (2016), reported a much higher accumulation of LREEs than of HREEs in various fish species in China, and these authors detected much more ∑REEs than ours. For example, Wang et al. (2022) determined ∑REEs ranging from 1.02 to 178 g g^−1^, with a mean concentration of 27.1 g g^−1^,almost twice our value (14.6 g g^−1^). This means that, in more polluted areas than ours, such as China, the predominance of LREEs can probably be expected.

### Accumulation of REEs and trace elements in tissues

**Fig. 2:**
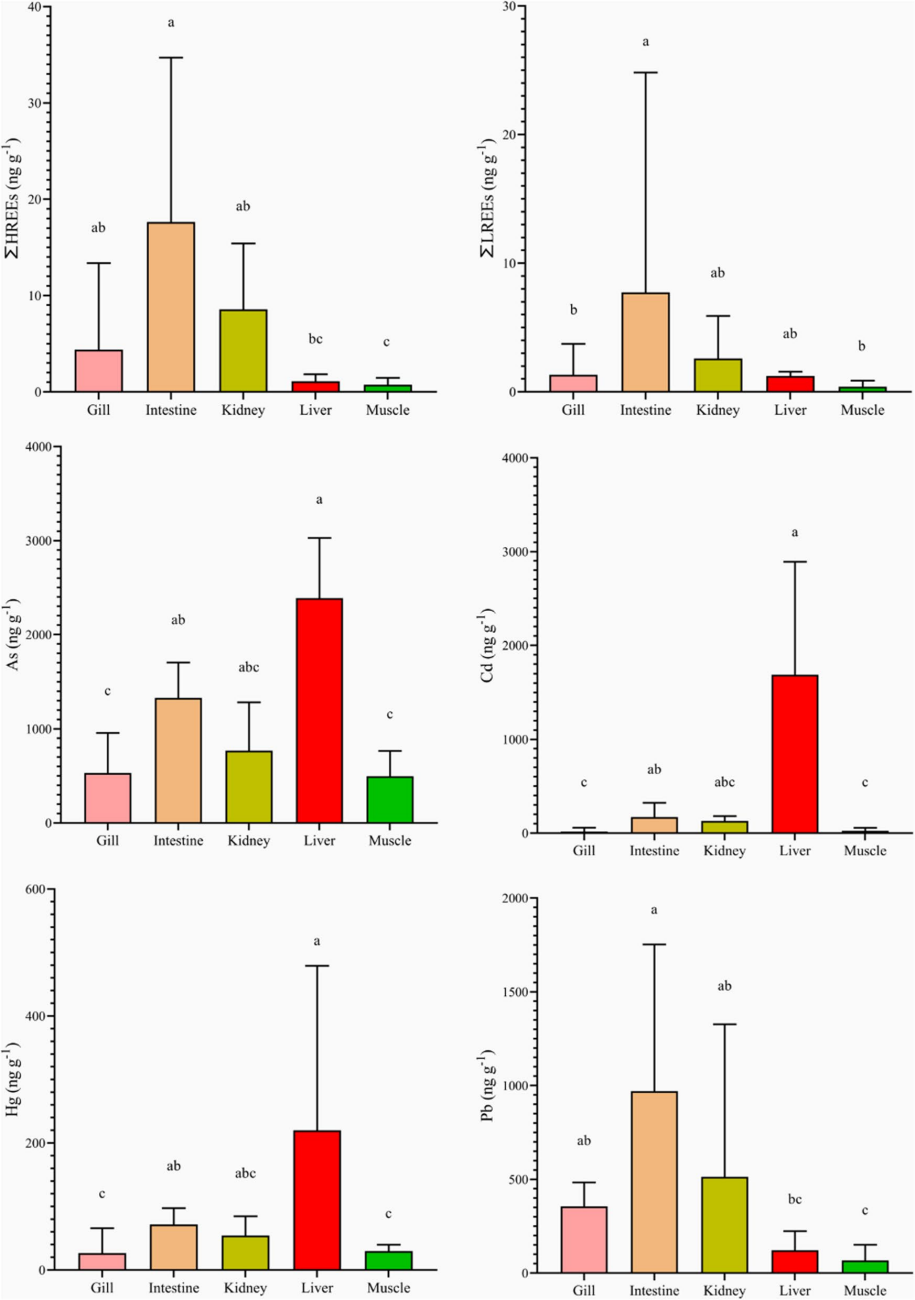
REEs, As, Cd, Hg and Pb accumulation patterns in different golden grey mullet (*Chelon auratus*) tissues. Data shown are medians and interquartile ranges

The medians and interquartile ranges of As, Cd, Hg, Pb and each REE, together with the ∑LREEs and ∑HREEs in the different golden grey mullet tissues, are shown in Table 2. With regard to the bioaccumulation patterns, we found that both the LREEs and the HREEs followed a similar trend, i.e. intestine > kidney > gills > liver > muscle. Small differences have been determined between some REEs. For example, Eu and La differed slightly from La, Ce, Pr, Nd and Sm, as seen in Table 2. Conversely, As, Cd and Hg mostly accumulated in the liver. Finally, Pb was accumulatecd in the tissues similarly to the REEs. The ∑LREEs, ∑HREEs and trace element accumulation patterns are displayed in Fig. 2. On the other hand, the ∑HREEs/∑LREEs calculated for each tissue were 3.28, 1.91, 3.31, 0.89 and 1.89 for gill, intestine, kidney, liver and muscle, respectively. This reflected that the HREE accumulation was three times that of LREEs in gills and kidney, and almost twice that in kidney and intestine. However, in the liver, LREEs were slightly more accumulated than HREEs.

**Table 2:** Median concentrations and interquartile ranges of the trace elements, ∑LREEs and ∑HREEs in different golden grey mullet (*Chelon auratus*) tissues

Element		Tissue				
		Gill	Intestine	Kidney	Liver	Muscle
Trace elements	As (ng g^−1^)	532^c^ (563)	1329^ab^ (829)	769^abc^ (638)	2389^a^ (1219)	497^c^ (367)
	Cd (ng g^−1^)	18.0^c^ (46.9)	172^ab^ (202)	130^abc^ (65.8)	1689^a^ (2098)	23.8^c^ (40.7)
	Hg (ng g^−1^)	26.5^c^ (55.2)	71.7^ab^ (35.3)	54.3^abc^ (32.3)	212^a^ (258)	29.6^c^ (15.9)
	Pb (ng g^−1^)	355^ab^ (235)	970^a^ (1312)	513^ab^ (932)	122^bc^ (113)	67.2^c^ (114)
LREEs	La (pg g^−1^)	206^b^ (500)	1290^a^ (2788)	445^ab^ (484)	211^ab^ (125)	59.6^b^ (115)
	Ce (pg g^−1^)	141^b^ (335)	1116^a^ (4573)	329^ab^ (312)	133^ab^ (76.7)	41.4^b^ (76.4)
	Pr (pg g^−1^)	73.2^b^ (166)	474^a^ (1027)	150^ab^ (169)	68.9^b^ (41.1)	22.5^b^ (40.9)
	Nd (pg g^−1^)	742^b^ (1654)	4996^a^ (10,503)	1380^ab^ (1835)	683^b^ (455)	225^b^ (384)
	Sm (pg g^−1^)	65.1^b^ (123)	415^a^ (865)	180^ab^ (161)	57.0^b^ (35.7)	18.4^b^ (27.4)
	Eu (pg g^−1^)	12.5^ab^ (17.5)	72.8^a^ (138)	21.8^ab^ (26)	9.33^b^ (6.80)	2.99^b^ (5.01)
	Sc (pg g^−1^)	93.6^ab^ (104)	533^a^ (1085)	85.7^ab^ (138)	43.0^b^ (27.8)	24.2^b^ (48.2)
	∑LREEs (ng g^−1^)	1.33^b^ (2.87)	9.24^a^ (2.07)	2.59^ab^ (3.12)	1.22^ab^ (7.43)	3.97^b^ (7.00)
HREEs	Gd (pg g^−1^)	118^b^ (235)	797^a^ (1619)	230^ab^ (279)	89.1^b^ (67.9)	36.7^b^ (54.9)
	Tb (pg g^−1^)	243^ab^ (453)	1636^a^ (3251)	488^ab^ (591)	160^b^ (104)	72.7^b^ (121)
	Dy (pg g^−1^)	87.5^ab^ (154)	556^a^ (1117)	164^ab^ (212)	56.5^b^ (43.7)	26.6^b^ (49.9)
	Ho (pg g^−1^)	6.98^ab^ (12.5)	47.2^a^ (92.6)	16.5^ab^ (20.2)	4.39^b^ (3.73)	2.23^b^ (3.32)
	Er (pg g^−1^)	25.2^ab^ (39.9)	154^a^ (301)	45.4^ab^ (59.3)	17.4^b^ (12.8)	7.14^b^ (11.9)
	Tm (pg g^−1^)	3.43^ab^ (4.18)	20.3^a^ (38.3)	4.93^ab^ (8.24)	3.30^b^ (2.64)	0.97^b^ (1.47)
	Yb (pg g^−1^)	0^ab^ (52.6)	88.2^a^ (153)	0^b^ (0)	0^ab^ (11.4)	0^b^ (0)
	Lu (pg g^−1^)	3.54^ab^ (3.25)	18.4^a^ (34.4)	5.91^ab^ (6.89)	3.45^b^ (2.39)	0.85^b^ (1.40)
	Y (pg g^−1^)	3897^ab^ (9146)	13992^a^ (20,380)	7231^ab^ (6076)	775^bc^ (658)	632^c^ (1064)
	∑HREEs (ng g^−1^)	4.38^ab^ (1.01)	17.6^a^ (2.74)	8.56^bc^ (6.66)	1.10^ab^ (1.00)	0.75^c^ (1.21)

^abc^Sharing at least one letter in a row indicates that there are no statistically significant differences

The high REE concentrations found in the intestine of the fish studied suggest that these elements are absorbed in small amounts by the intestinal mucosa. In fact, several studies have reported that only very small amounts of REEs ingested orally are absorbed in the gastrointestinal tract (Damment & Pennick, 2007; Fricker, 2006). Regarding the toxicokinetics, REEs are accumulated to a greater degree in organs, viscera and bone, compared to in muscle tissues in pelagic fish (Mayfield & Fairbrother, 2015). The REE absorption in fish is always lower than in plants and in benthic organisms (Mayfield & Fairbrother, 2015; Squadrone et al., 2019).

In another sense, the differences between tissues could be explained in terms of the function of each organ (excretion and detoxification), and the different metabolic functions of the trace elements, for which higher concentrations in the liver and kidney than in the muscle are generally reported (Squadrone et al., 2022). Although the presence of REEs in fish is little studied, our results indicated that they are indeed accumulated in very small amounts in the muscle, the same as metals like As, Cd, Hg and Pb. However, despite muscle accumulation being lower compared with that in other tissues, the Cd concentrations, mainly, and to a lesser extent the Pb ones, exceeded the maximum limits permitted by the European Union for these metals (50 ng g^−1^ for Cd and 200 ng g^−1^ for Pb) (European Commision, 2006), since the maximum values for these metals in muscle were of 679 and 262 ng g^−1^, respectively.

As argued by Amyot et al. (2017), the fact that muscle tissue accumulates fewer elements is positive for human health, because it is the main part of the fish consumed by people. However, from an ecotoxicological perspective, both REEs and heavy metal concentrations are much higher in the viscera, which means that fish predators, which consume the whole animal, are probably exposed to much higher doses. However, when the concentrations in muscle by itself exceed the maximum limits permitted in fish, as in this case, it is a sign that the ecosystem is really exposed to high doses of metals.

## Conclusions

The HREE levels in golden grey mullet were higher than those of LREEs, whereas, actually, the latter are infinitely more abundant in the environment. The levels of Tb, a very scant element, were particularly high in the samples analysed, which infers that the area is contaminated by the oil refinery.

The intestine tissue gave the highest REE concentrations, which may be indicative of a very low gastrointestinal absorption. The lowest concentrations out of all the elements analysed were found in the muscle tissue, which could be a positive result for humans as it is mainly the part consumed.

Lastly, the fact that the Cd and Pb levels in muscle exceed the maximum limits permitted is of great concern, requiring some measures or regulations to be adopted in the area, such as neutralizing the dumping of waste water, or making the population aware of the excessive use of chemical products, in addition to going on investigating the ecotoxicity of these contaminants.

## Data Availability

The data that support the findings of this study are available from the corresponding author.

## Abbreviations

REEs: Rare earth elements
LREEs: Light rare earth elements
HREEs: Heavy rare earth elements
